# Supplementary material for: Improving Agrobacterium tumefaciens−Mediated Genetic Transformation for Gene Function Studies and Mutagenesis in Cucumber (Cucumis sativus L.)
Source: Genes (Basel). 2023 Feb 27;14(3):601. doi: 10.3390/genes14030601 (PMC10048676; doi:10.3390/genes14030601)
Supplement: Supplementary file 1 [file genes-14-00601-s001.zip › Supplemental file S1.pdf]

Supplemental File S1

Protocol of *Agrobacterium tumefaciens*-mediated Genetic Transformation in Cucumber

(Cucumber Improvement Program, USDA-ARS,
Horticulture Department, University of Wisconsin Madison)

Note: This protocol is optimized for the cucumber line Poinsett 76. The overview of the whole cycle of the transformation process is illustrated in Appendix I which could be completed in ~four months. The formulae of various growth media are provided in Appendix II. Chemicals and preparation of stock solutions are detailed in Appendix III.

Seed sterilization

1. Select 100 full seeds (Poinsett 76) and put them in a 50-mL tube with cap. Soak seeds in a water bath at 55 ℃ for 15 min, and then continues to soak for 2h at 25℃.
2. Peel off seed coat with forceps. This could be performed on a lab bench.
3. Surface sterilize the seeds by soaking them in 75% ethanol for 1 min in a laminar flow hood. Gently shake the tube while soaking. Note that operations from Steps 3 to 24 below need to be conducted in a sterilized environment such as a laminar flow hood.
4. Rinse the seeds once with sterile ddH_2O. Decant the water.
5. Sterilize the seeds in 6% NaOCl for 12 min. Mix the seeds well by gentle shaking of the tube.
6. Decant the NaOCl solution. Rinse the seeds three times with sterile ddH_2O.
7. Blot dry seeds on sterile filter papers in a large sterile Petri dish.

Explant preparation

8. Place 25 sterilized seeds in a Petri dish (100×15 mm) filled with 25 ml MGM. Seal the dish with parafilm and incubate at 28 ℃ in dark for 72h (Figure 1A). The time of incubation may need to be optimized which may depend on genotype, seed age, or maturity.
9. Dissect two cotyledons from the germinating seed with sterilized forceps and a scalpel. Remove the portion containing shoot apical meristem (SAM) cells on the cotyledon. Cut off the cotyledons in the middle transversely. Discard the distal half. Harvest the proximal half (hypocotyl end, with subapical ground meristem tissue) of the cotyledon as the explant. Two cotyledon explants could be harvested from one seed. Place the explants promptly into a Petri dish containing 15 ml MLM to keep explants wet and fresh (Figure 1B).
10. If seed availability is of concern or more explants are desired, the proximal half of cotyledons can be cut further in the middle longitudinally. The proximal part of the hypocotyl can also be dissected as explant. Up to five explants (four from two cotyledons and one from the hypocotyl) could be harvested from one seed (Figure 1C).

Agrobacterium inoculum preparation

11. One day before seed germination, streak one loopful of *Agrobacterium* stock culture (AGL1 strain in our protocol) onto the solid LB media containing 50 mg/L carbenicillin (or 50 mg L^{-1}

rifampicin) and 50 mg/L kanamycin (or other antibiotics as appropriate). Incubate in an oven at 28-30℃ for 48h.

12. Streak a single colony of *Agrobacterium* and resuspend in 10 µL distilled water. Perform PCR and DNA sequencing of the target gene to confirm that the bacterium carries the correct construct. Once confirmed, store the LB plate at 4 ℃ for use (up to one month).
13. Scrap a single colony of *Agrobacterium* off the LB plate and resuspend in 10 ml liquid LB in a conical flask. Culture the suspension in an incubator shaker (28 ℃ at 200 rpm) overnight. Transfer 200 µL of the cultured suspension into a new conical flask containing 50ml fresh LB. Place it in a incubate shaker (28 ℃ at 200 rpm) for ~10h until the OD_{600} reaches 0.7. Centrifuge the suspension at 3000 rpm for 5 min. Discard the supernatant. Resuspend the cells in 20 mL sterile MLM. Adjust OD_{600} to 0.7. Add acetosyringone (final concentration 200 µM) and Silwet L-77 (final concentration 0.05%) to the suspension. For 100 seeds, ~20 mL of suspension is needed (Figure 1D). Keep the bacterium suspension at 28 ℃ until use.

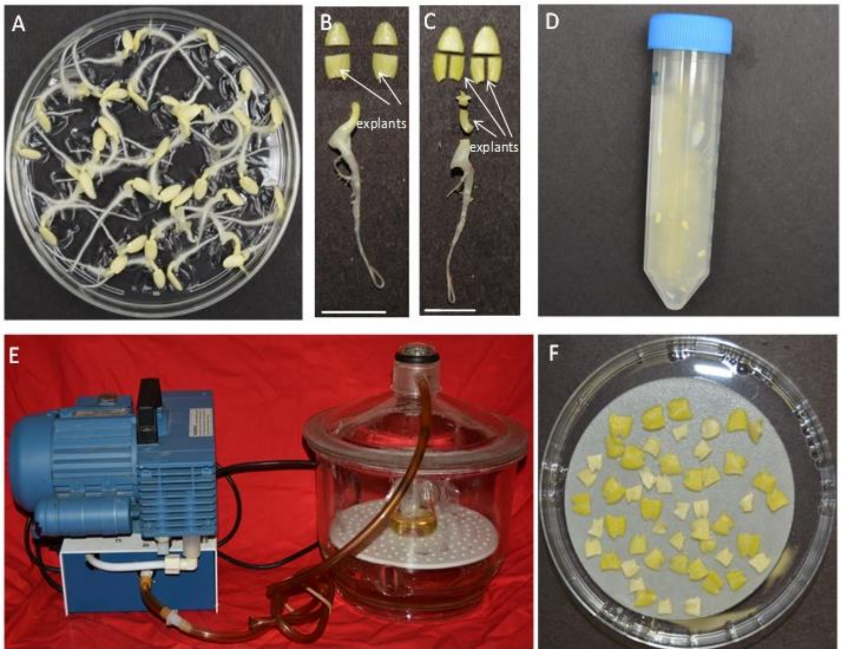

Figure 1. Preparation of explants and *Agrobacterium* inoculation. (A) Germinating seeds on MGM for 72h in dark at 28℃. From one seed, 2 or 5 explants (B-C) could be harvested. (D) *Agrobacterium* suspension in a 50-ml centrifuge tube for inoculation. (E) A system for vacuum infiltration for *Agrobacterium* infection of explants. (F) Explants after co-cultivation for 48h. Note the presence of white and yellow colored explants. Only yellow explants have the ability to regenerate shoots.

***Agrobacterium* inoculation and vacuum infiltration**

14. When both explants and *Agrobacterium* inoculum are ready, immediately transfer the explants to the flask containing the *Agrobacterium* suspension. Cover the flask with semipermeable film.
15. Incubate the explants in the *Agrobacterium* suspension for 12 minutes at room temperature with gentle shaking. Optionally, one could put the flask in a vacuum (Figure 1E). Run the vacuum at 0.07 mpa for 12 min at room temperature. Vacuum infiltration has been proposed to enhance bacterial infection. This procedure may increase the chance of contamination.
16. Pour off the *Agrobacterium* suspension. Dump the explants onto the sterile filter paper in a large Petri dish. Caution! Do not rinse the explants with sterile water at this step. Blot explants to remove excessive *Agrobacterium* suspension.

***Agrobacterium*-explant co-cultivation**

17. Place one sterile Whatman filter paper (7 cm diameter) in a Petri dish (100 ×15 mm) with solid CMM. Put ~40 explants into each dish. Spread the explants evenly to lightly cover the filter paper (Figure 1F).
18. Incubate the Petri dish in dark at 23℃ for 48h.

Shoots initiation and regeneration

19. Rinse the explants three times in MLM after co-cultivation for 48h. Pour off the MLM. Blot dry the explants with sterile filter papers.
20. There are two types of explants with whitish or yellowish color (Figure 2A-B). Discard the whitish ones. Transfer the healthy yellowish explants to SIM containing 200 mg/L Timentin to kill/inhibit *Agrobacterium* and appropriate selective agent (100 mg/L kanamycin for Poinsett 76) to kill untransformed cells. Place ~15 explants per Petri dish (100×25mm).
21. Incubate at 28°C/18°C with a 16h/8h light/dark temperature/light cycle. Shoots usually will be visible in 7-10 d (Figure 2C). It takes ~ 2 weeks for the shoot to be ready for subculture (Figure 2D).
22. Dissect the green shoots from the explants (one shoot from one explant) and place them in a jar containing fresh SIM with 200 mg/L Timentin and appropriate selective agent for subculture. Discard shoots showing chlorosis (Figure 2E-F).
23. Subculture the green shoots/plantlets every two weeks in SIM until the plantlets are large enough for rooting (Figure 2F).

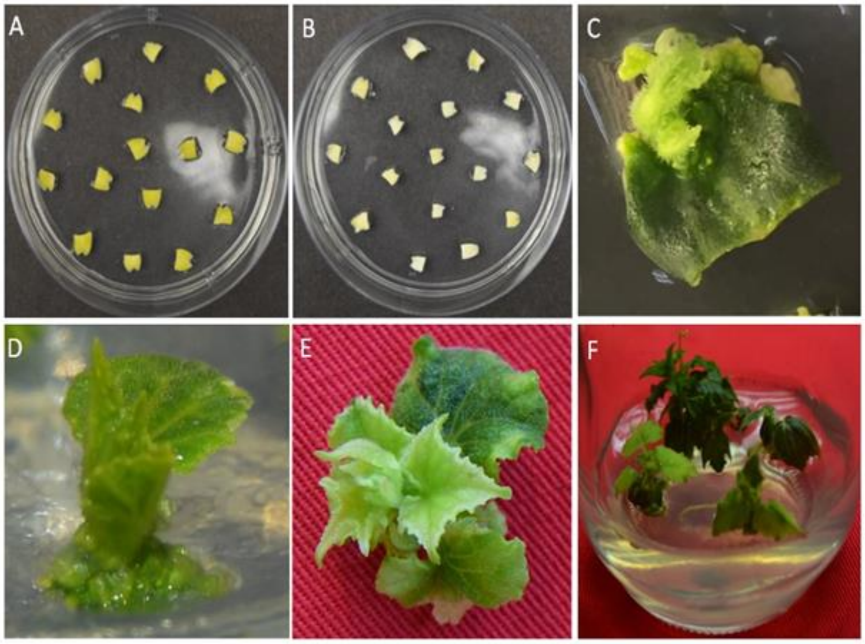

Figure 2. Shoot initiation and regeneration from explants. (A-B). Co-cultivation with *Agrobacterium* may yield yellowish (A) and whitish (B) explants. (C). Shoots growing out of the yellow explants are visible in approximately two weeks. (D). Subculture of the green shoots. (E-F). Shoots or plantlets regenerated may be healthy green or chlorotic.

Rooting, plant regeneration, and validation

24. Transfer the shoots into a jar with full strength RMM. Use half strength RMM if the plantlets are difficult to root (Figure 3A-D).
25. When a plantlet produces at last 3 roots (≥ 2 cm) (Figure 3C), carefully transplant it into soil in a plastic cube (7×7 cm). Cover the plantlet with a plastic dome or saran wrap film (Figure 3E) to maintain humidity. Adjust the temperature and light conditions for easy acclimation. Don't remove the dome/film wrap until the plantlet regains growth (usually in 3-5 days). After ~one-week of training, transfer the plantlet to a large pot, and move the pot into a greenhouse.
26. When the plant reaches around five-true-leaf stage (Figure 3F), collect samples from young leaves for DNA extraction and PCR validation (Figure 3G). Note that sample old tissues may increase the chances of false positives caused by residual *Agrobacterium* cells on old leaves. Keep PCR-positive plants only. Discard PCR-negative plants to save time and space.
27. Make self-pollinations to advance T_0 plants to T_1, or other crosses on putative transgenic plants (Figure 3H).

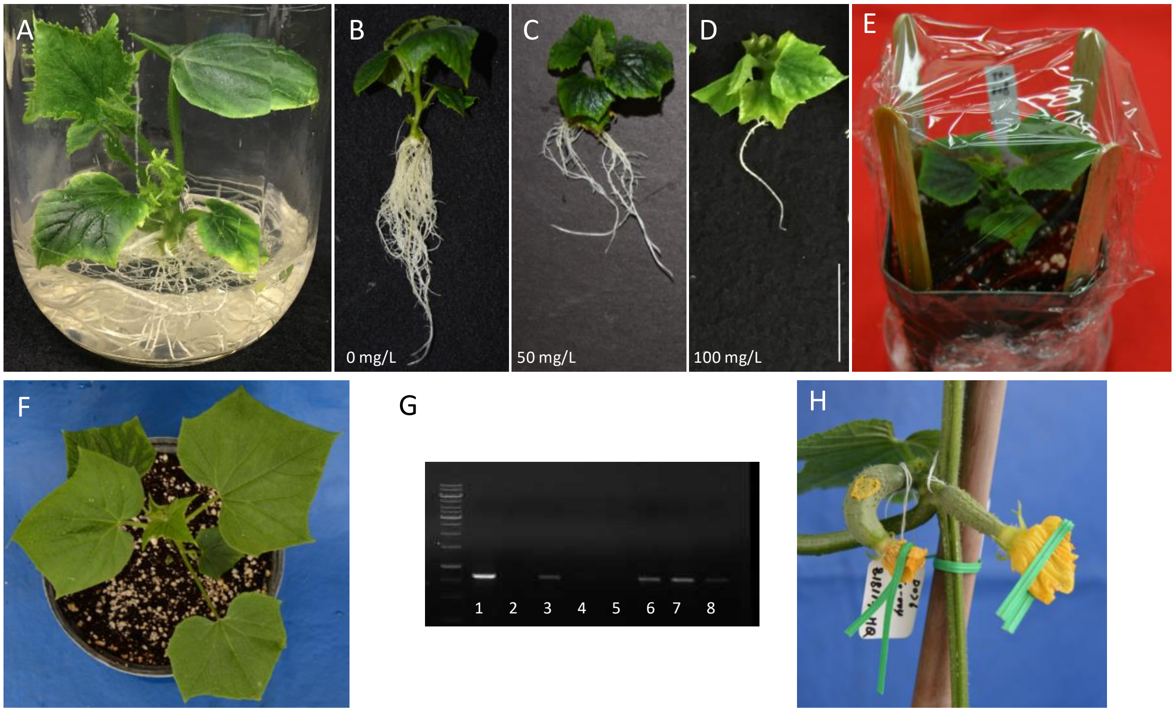

Figure 3. Roots initiation and PCR assays. (A) After placing in rooting MS medium (RMM) for 2 weeks, roots are clearly visible. (B-D) Roots from RMM containing 0, 50 and 100 mg/L kanamycin as selective agent. (E) The plantlet transplanted from the jar is covered under a saran wrap film to maintain humidity. (F) Putative transgnic seedling at 5-real-leaf stage. (G) Validation of putative T_0 transgenic plants by PCR of a T-DNA vector specific DNA sequence (GUS gene in this example). The first lane is size marker, and Lanes 1-8 are positive control and 7 regenerated plantlets from independent transformation events, respectively. (H) Generate seeds from T_0 plants through self-pollination.

Appendix I. Flowchart and approximate timeline for a typical transformation cycle.

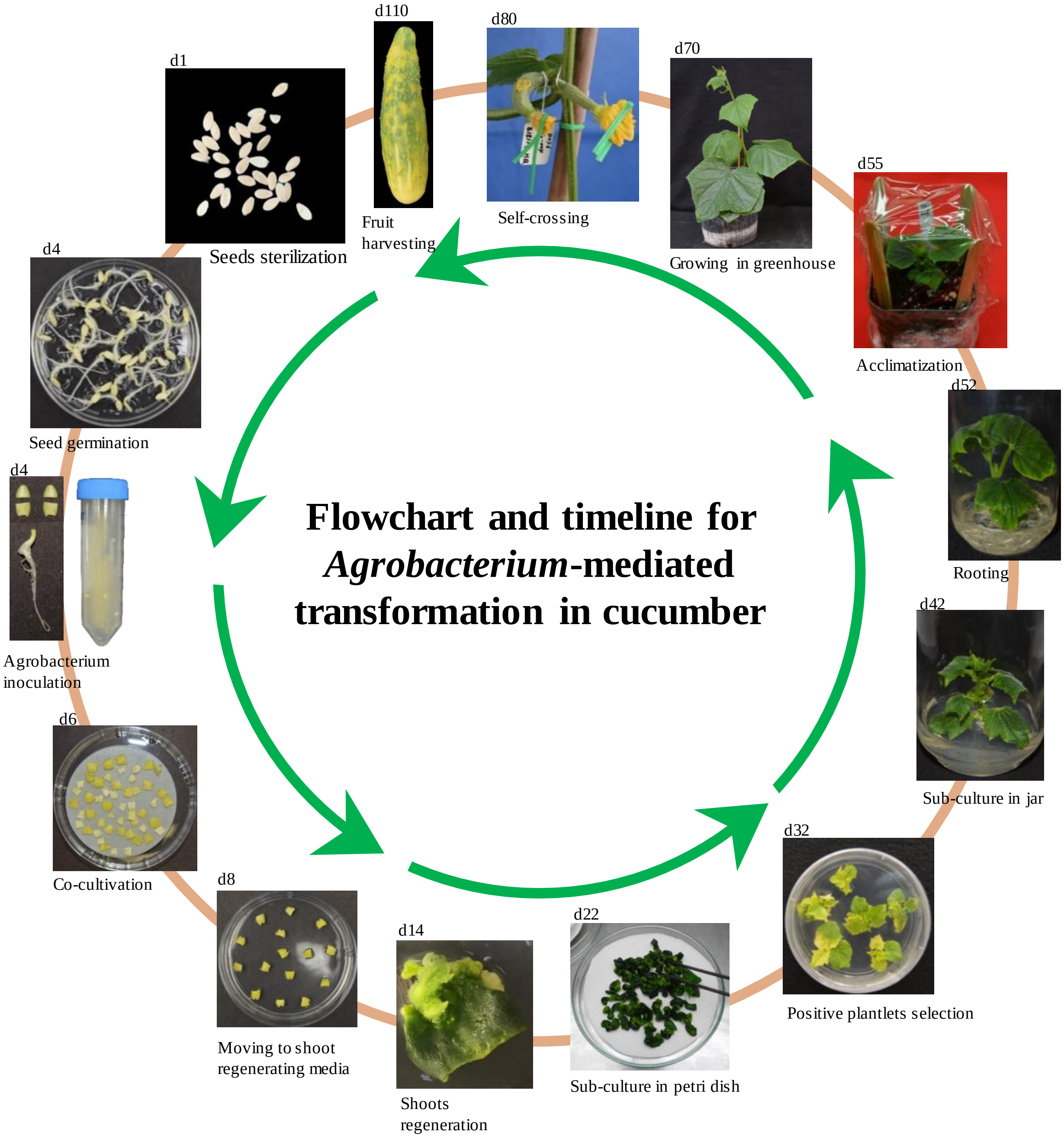

Appendix II. Culture media for *Agrobacterium*-mediated tranformation

½ MS germination medium(MGM, per 100ml)

MS powder	0.22 g
Sucrose	3.0 g
Phytagel	0.26 g
H_2O to	100 mL
pH	5.8 (Adjust with 1.0 M NaOH)

½ MS liquid medium(MLM, per 100ml)

MS powder	0.22 g
Sucrose	3.0 g
H_2O to	100 mL
pH	5.8 (adjust with 1.0 M NaOH)

Co-culture MS medium(CMM, per 100ml)

MS powder	0.44 g
Sucrose	3.0 g
Phytagel	0.26 g
6-BA	Final concentration to 1.0 mg/L
ABA	Final concentration to 0.5 mg/L
H_2O to	100 mL
pH	5.8 (adjust with 1.0 M NaOH)

Shoots initial medium(SIM, per 100 ml)

MS powder	0.44 g
Sucrose	3.0 g
Phytagel	0.26 g
6-BA	Final concentration to 1.0 mg/L
ABA	Final concentration to 0.5 mg/L
Kanamycin	Final concentration to 100 mg/L
Timentin	Final concentration to 200 mg/L
H_2O to	100 mL
pH	5.8 (adjust with 1.0 M NaOH)

Rooting MS medium (RMM, per 100ml)

MS powder	0.22 g
Sucrose	3.0 g
Phytagel	0.26 g
Kanamycin	Final concentration to 50 mg/L
Timentin	Final concentration to 200 mg/L
H_2O to	100 mL
pH	5.8 (adjust with 1.0 M NaOH)

Solid LB medium (per 100ml)

LB Broth	2.0 g
Agar	1.5 g
H_2O to	100 mL

Liquid LB medium (per 100ml)

LB Broth	2.0 g
H_2O to	100 mL

Appendix III. Chemicals and stock solution preparation for Agrobacterium-medied transgeformation in cucumber.

Reagents	Stock solution preparation	Stock concentration	Sterilization	Vendors	CAT #
Murashige and Skoog (MS)	-	-	Autoclave	PhytoTech Labs	M524
Gellan Gum, CultureGel (Phytagel)	-	-	Autoclave*	PhytoTech Labs	G434
Sucrose	-	-	Autoclave	Thermo Scientific	AA36508A1
LB Broth	-	-	Autoclave	Fisher BioReagents	BP1426-2
Sodium Hypochlorite	-	-	No need	Fisher Chemical	SS290-1
6-Benzylaminopurine (6-BA)	Dissolve 0.02 g 6-BA in 5 mL of 1 mol/L NaOH, then add water to 10 mL	2 mg/mL	Autoclave or 0.22 µm Sterile filter	MP Biomedicals	MP021009125
Abscisic Acid (ABA)	Dissolve 0.01 g ABA in 5 mL absolute ethanol, then add water to 10 mL	1 mg/mL	0.22 µm Sterile filter	Thermo Scientific	AC133485000
Acetosyringone (AS)	Dissolve 196 mg Acetosyringone in 5 mL methanol, then add water to 10 mL	100 mM	0.22 µm Sterile filter	TCI America	D26665G
Kanamycin (KAN)	Dissolve 500 mg kanamycin in10 mL water	50 mg/mL	0.22 µm Sterile filter	Thermo Scientific	AC450810100
Chloramphenicol	Dissolve 250 mg chloramphenicol in10 mL absolute ethanol	25 mg/mL	0.22 µm Sterile filter	Thermo Scientific	AC227925000
Timentin	Dissolve 2 g timentin in10 mL water	200 mg/mL	0.22 µm Sterile filter	Bioworld	NC9734923
Silwet L-77	-	Original	0.22 µm Sterile filter	Bioworld	NC1791615
Carbenicillin	Dissolve 0.5 g carbenicillin in10 mL water	50 mg/mL	0.22 µm Sterile filter	Fisher BioReagents	BP2648250
Hygromycin (HGY)	Dissolve 500mg hygromycin in 10 ml H2O	50mg/ml	0.22 µm Sterile Filter	MP Biomedicals	ICN19417083

***** Phytagel is not re-autoclavable.
