# Supplementary material for: Improving Agrobacterium tumefaciens−Mediated Genetic Transformation for Gene Function Studies and Mutagenesis in Cucumber (Cucumis sativus L.)
Source: Genes (Basel). 2023 Feb 27;14(3):601. doi: 10.3390/genes14030601 (PMC10048676; doi:10.3390/genes14030601)
Supplement: Supplementary file 1 [file genes-14-00601-s001.zip › Supplemental_table and figures_R1.pdf]

Table S1. Transformation efficiency (TE) of different genotypes from various experiments.

Genotype	Gene	*Agrobacterium* strain	Transgene approach	# Seeds tested	# Explants used	# transformant*	TE (%) by seeds	TE (%) by Explants
**PS76**	*LL*	AGL1	Overexpressing	2103	4925	24	1.14	0.49
	APRR2	AGL1	RNAi	500	1000	3	0.60	0.30
	APRR2	GV3101	RNAi	500	1000	1	0.20	0.10
	Mean						0.90	0.40
**H19**	*LL*	AGL1	Overexpressing	200	400	1	0.50	0.25
**9930**	*FS1.1*	AGL1	Overexpressing	600	1200	3	0.50	0.25
**9930**	*Cca4*	AGL1	Overexpressing	300	1200	5	1.67	0.42
**9930**	*SGR*	EHA105	CRISPR-Cas9	2050	4100	23	1.12	0.56
	Mean						1.05	0.48

* Validated with various methods

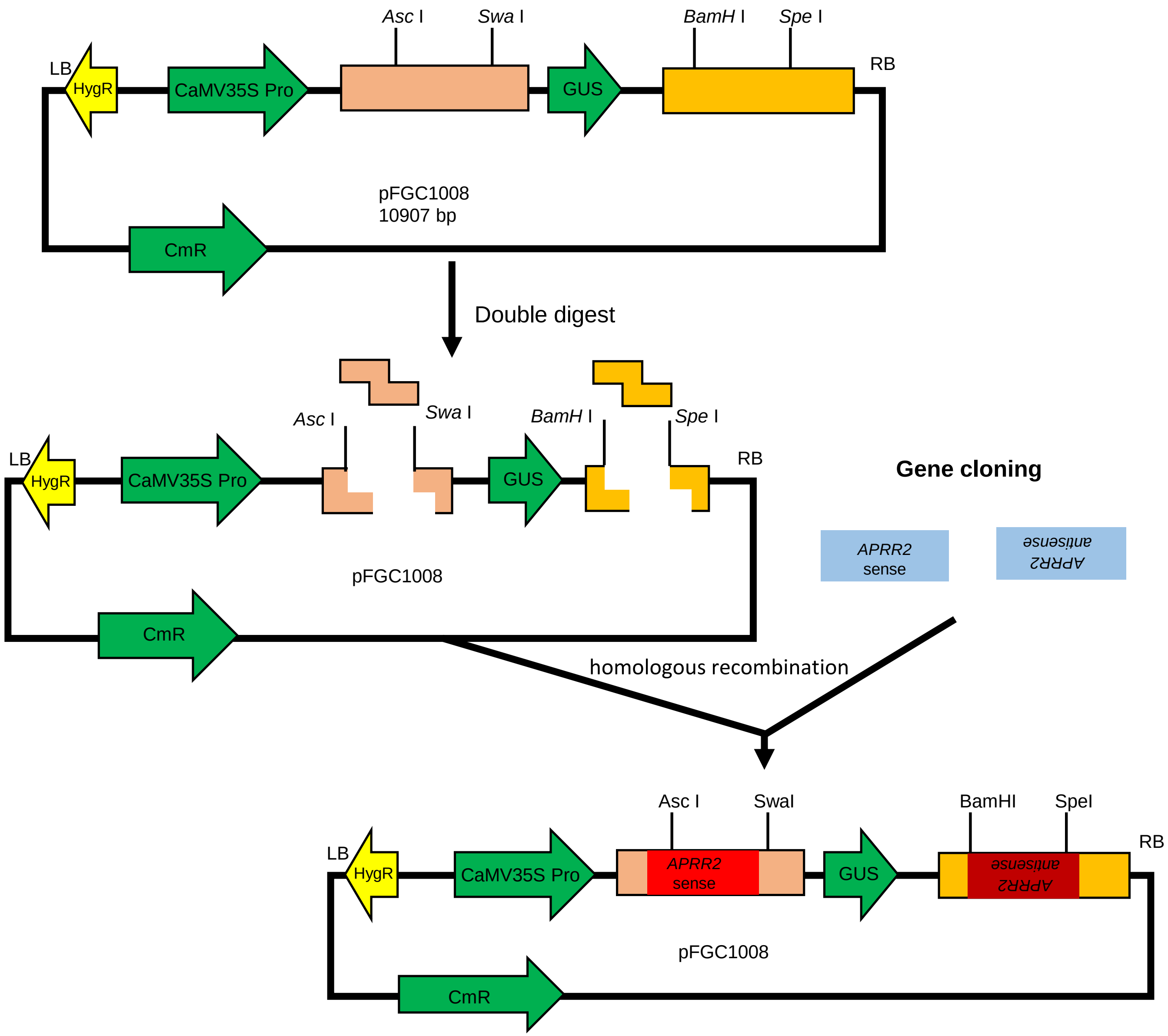

Figure S1 RNAi construction of *APRR2* in pFGC1008. Two *APRR2*-specific fragments were cloned and inversely inserted into the pFGC1008 plasmid double digested with *Asc I*/*Swa* I and *BamH* I/*Spe* I. *HypR* is selective marker gene for hygromycin. CmR is selective marker gene for chloramphenicol. Pro indicates promoter. GUS is reporter gene for β-glucuronidase. LB and RB represent left and right borders of the T-DNA**. Primer sequences for** APRR2-sense are Forward (5'→3'): ACAATTACCATGGGGCGCGCCTGGCTTACCCTTCTTATCAT; Reverse (5'→3'): TTCATCTGGGGATTTAAATGGACGGTGGAGATTCCTTT; for APRR2-antisense Forward (5'→ 3'): GATCTCTTTGATGGGGATCCGGACGGTGGAGATTCCTTT, Reverse (5'→3'): GCAGGACTCTAGGGACTAGTTGGCTTACCCTTCTTATCAT.

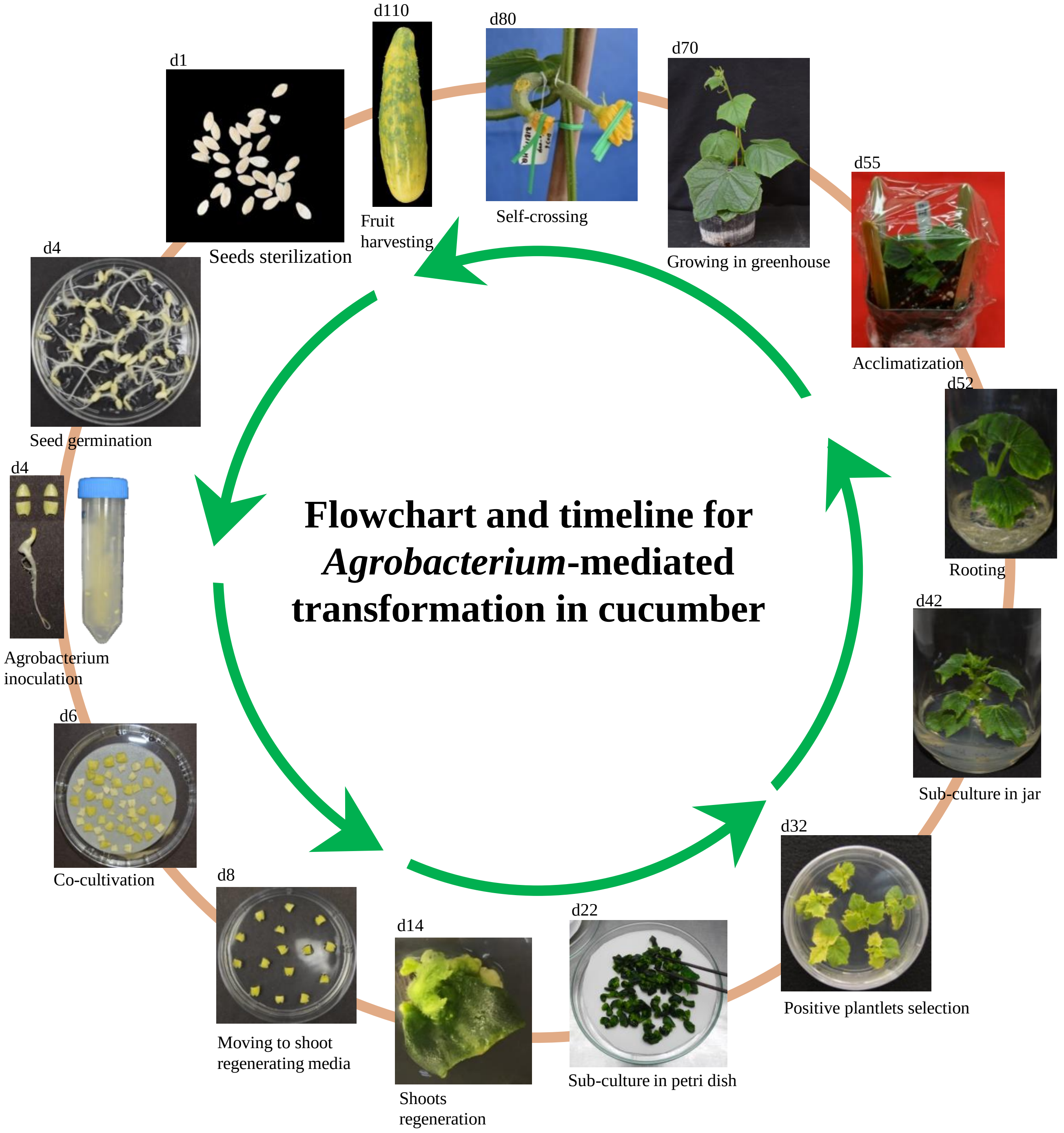

Figure S2. Life cycle of *Agrobacterium*-mediated transformation in 110 days in cucumber. Fourteen main steps were included in this 110-day of transformation protocol, which started from sterilizing seeds, and ended at extracting T_1 seeds from T_0 fruit.

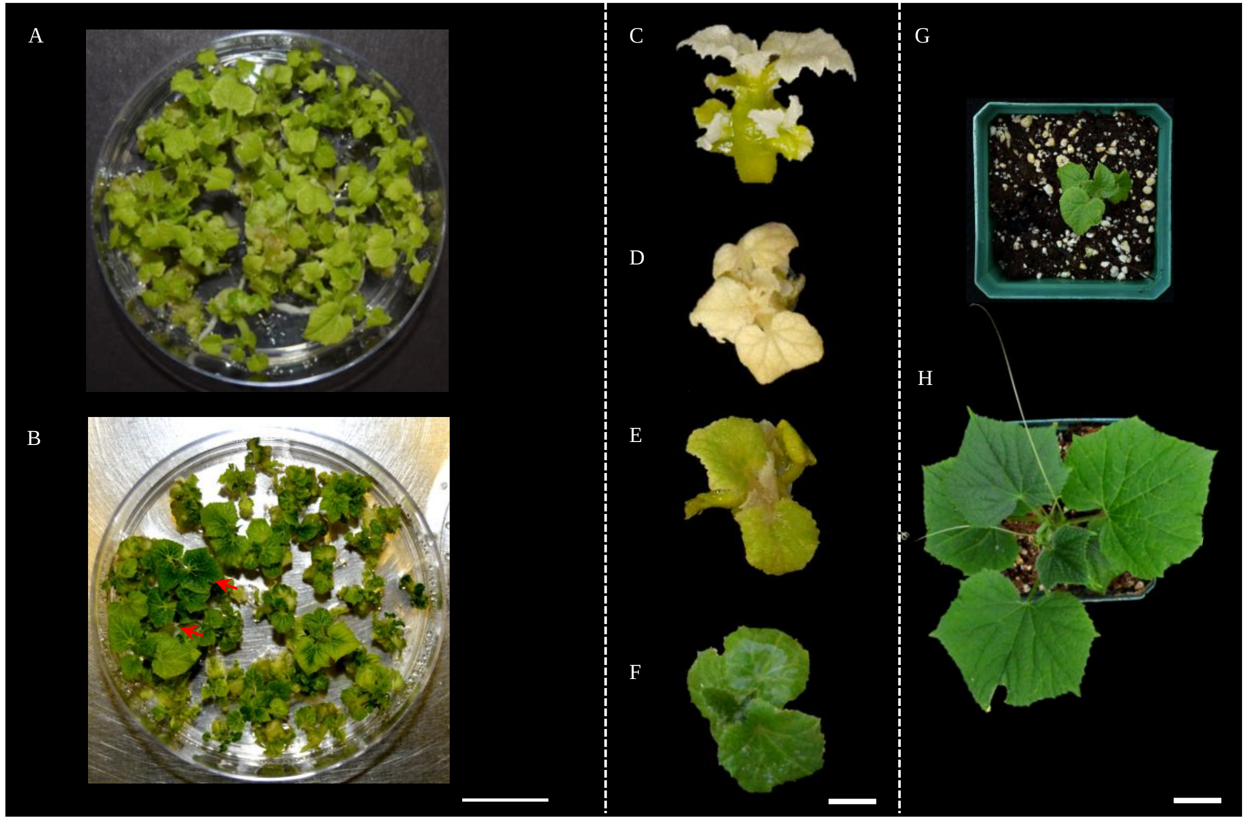

Figure S3. Development of transgenic plant overexpressing the *LL* gene in PS76 using hypocotyl as the explant. A. Shoots generated from SAM. **B.** Shoots generated from hypocotyl. **C-F.** Shoots regenerated in growth media selected at 100 mg/L of kanamycin. Only true transformants will survive (**F**). Leaves could be differentiated from the middle and top section of the hypocotyl (**C**). **G** Plantlet is transplanted to soil. **H** T_0 plant is growing in the greenhouse. Bar = 1.0 cm.

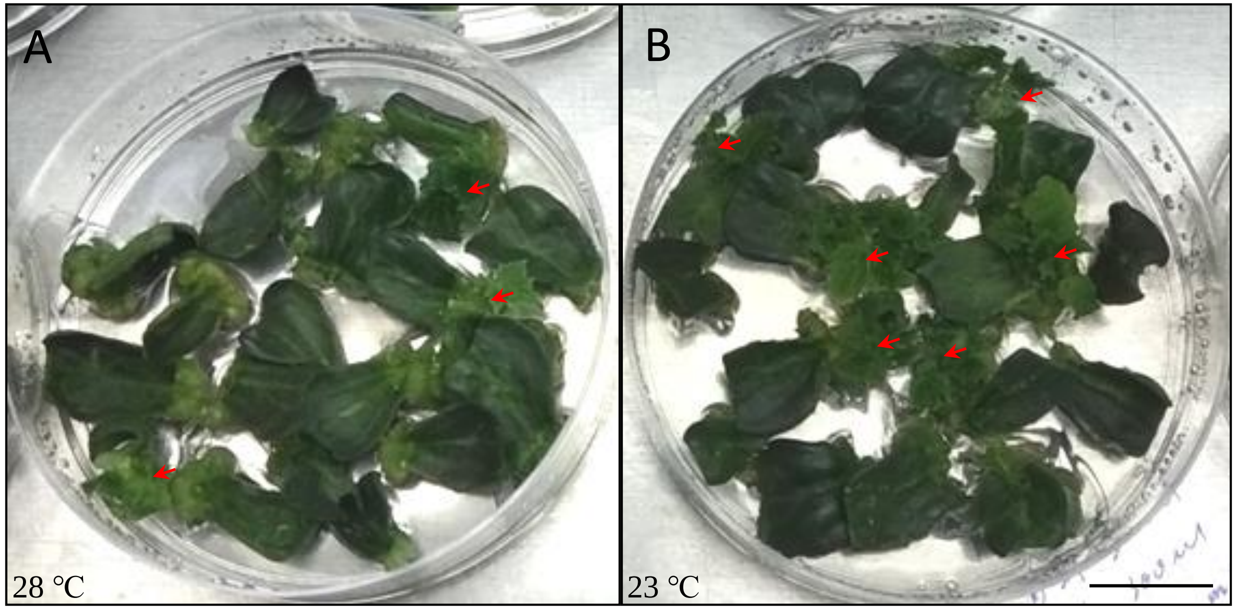

Figure S4. Shoot regeneration from explants co-cultured with *Agrobacterium* at 28 °C (A) and 23 °C (B) with 50 mg L^{-1} kanamycin added in the MS media. The red arrows indicate regenerated shoots. Bar = 1.0 cm.

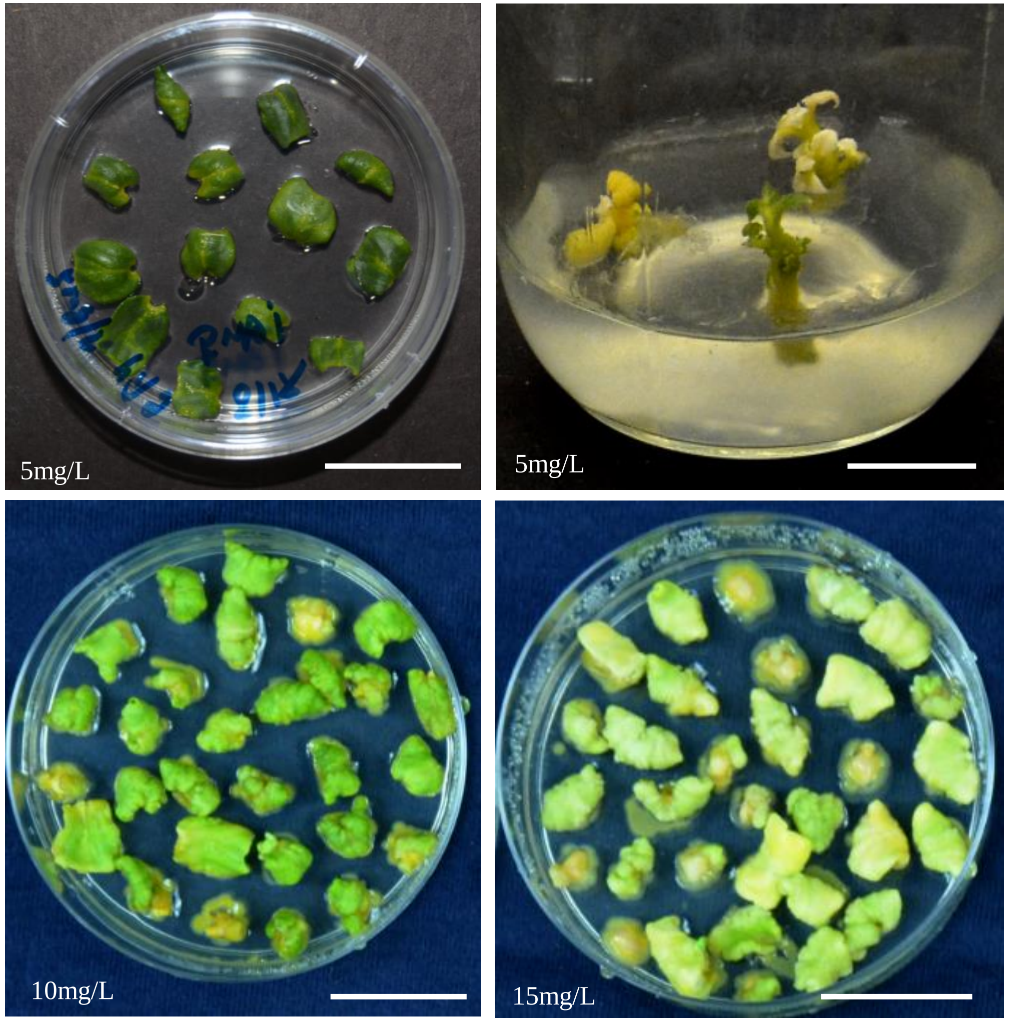

Figure S5. Effect of concentration of hygromycin on survival of explants and shoot regeneration. Explants are gown in MS media with 5, 10 and 15 mg L^{-1} hygromycin. Bar = 1.0 cm.

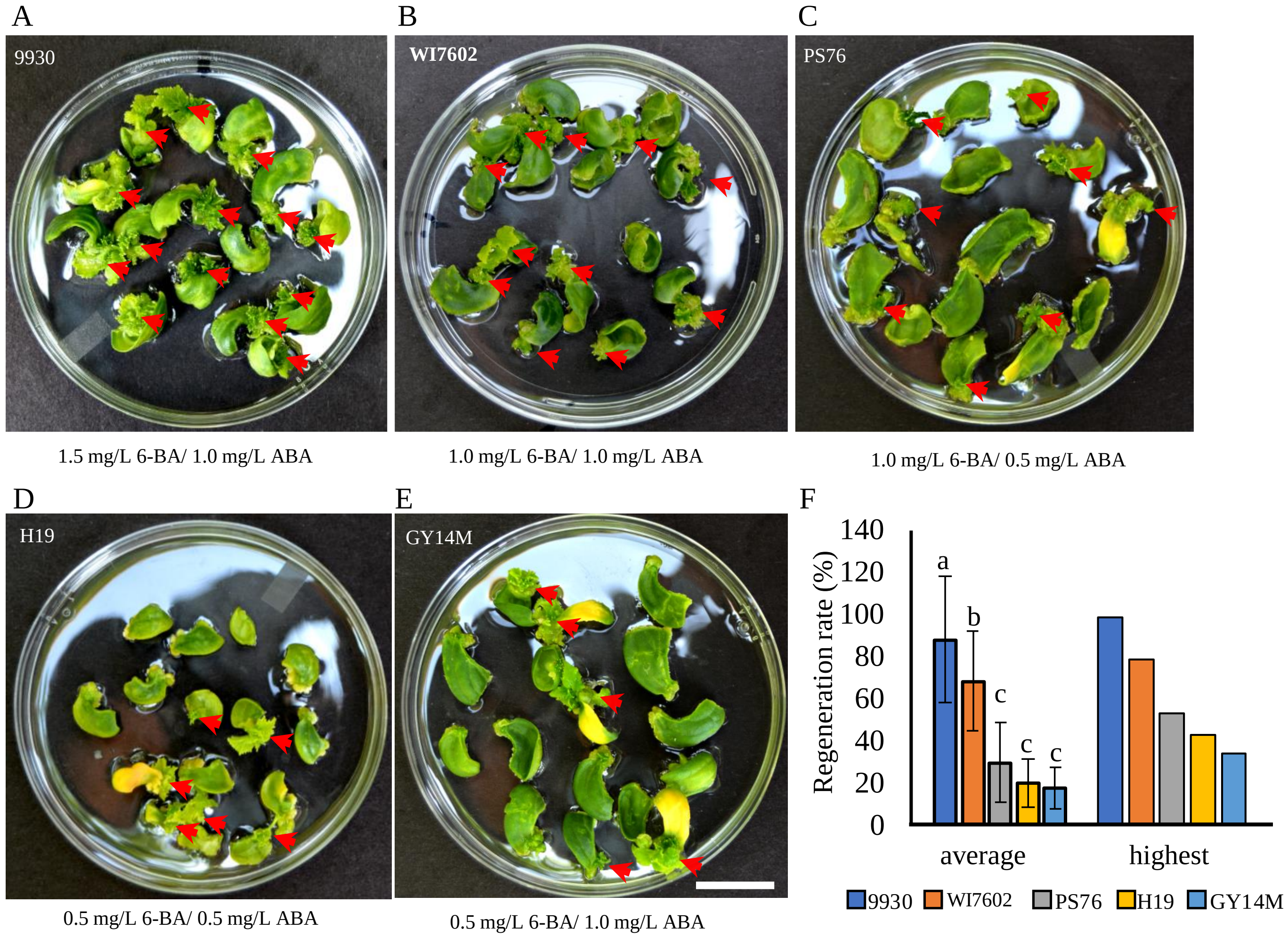

Figure S6. The highest regeneration frequency at the given hormone concentration in 5 tested cucumber genotypes. A-E. Represented pictures for explants generating shoots. **F.** The average and highest regenerating rate for each line. Values are means ± SD. The letters a–c indicate statistically significant differences between means of shoot regeneration rate based on one-way ANOVA analyses with Tukey HSD test ($P < 0.05$). The red arrows indicate the regenerated shoots. Bar = 1.0 cm.

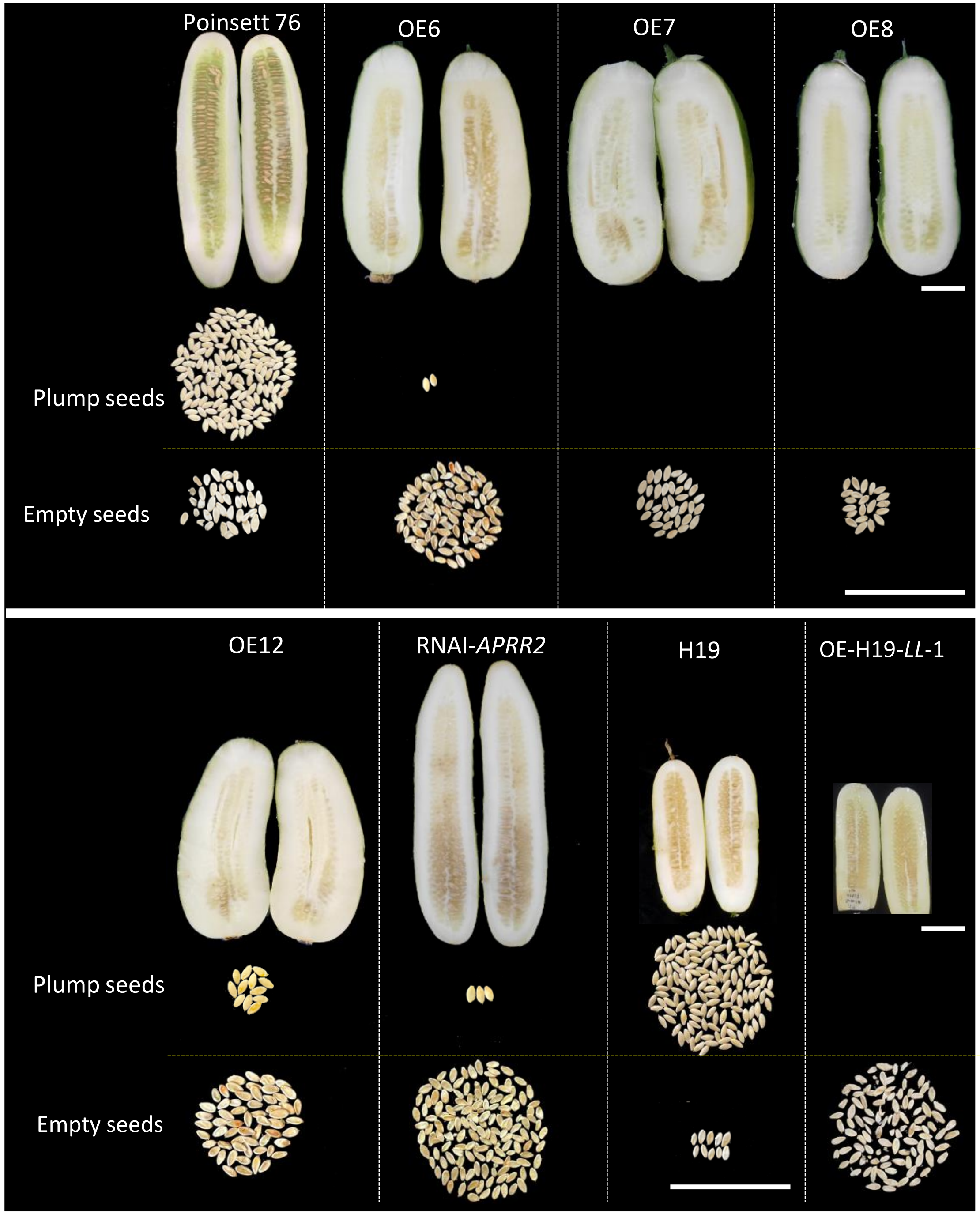

Figure S7. Lower fertility in T_0 transgenic OE and RNAi plants obtained from the present study. Poinsett 76 and H19 can set many well-developed plump seeds with very few empty seeds. Fruits from very few OE or RNAi plants have very few well-developed seeds; some are sterile with no seeds. Bar = 5.0 cm.

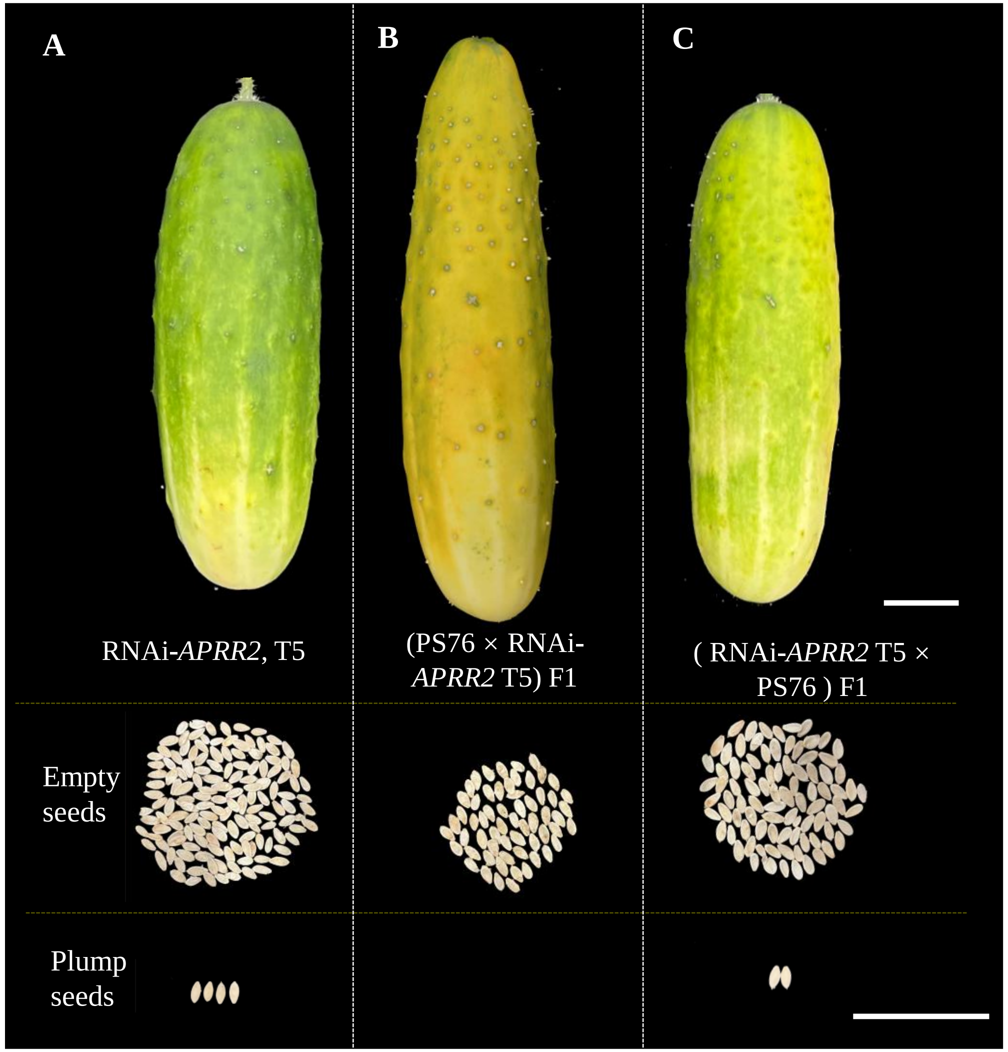

Figure S8. Lower fertility remains in T_5 transgenic plants of PS76-RNAi-*APRR2*. Low seed set in T_5 transgenic plants (**A**) and the reciprocal F_1 from the crosses between RNAi-*APRR2*. T_5 and PS76 (**B** and **C**). Bar = 5.0 cm.

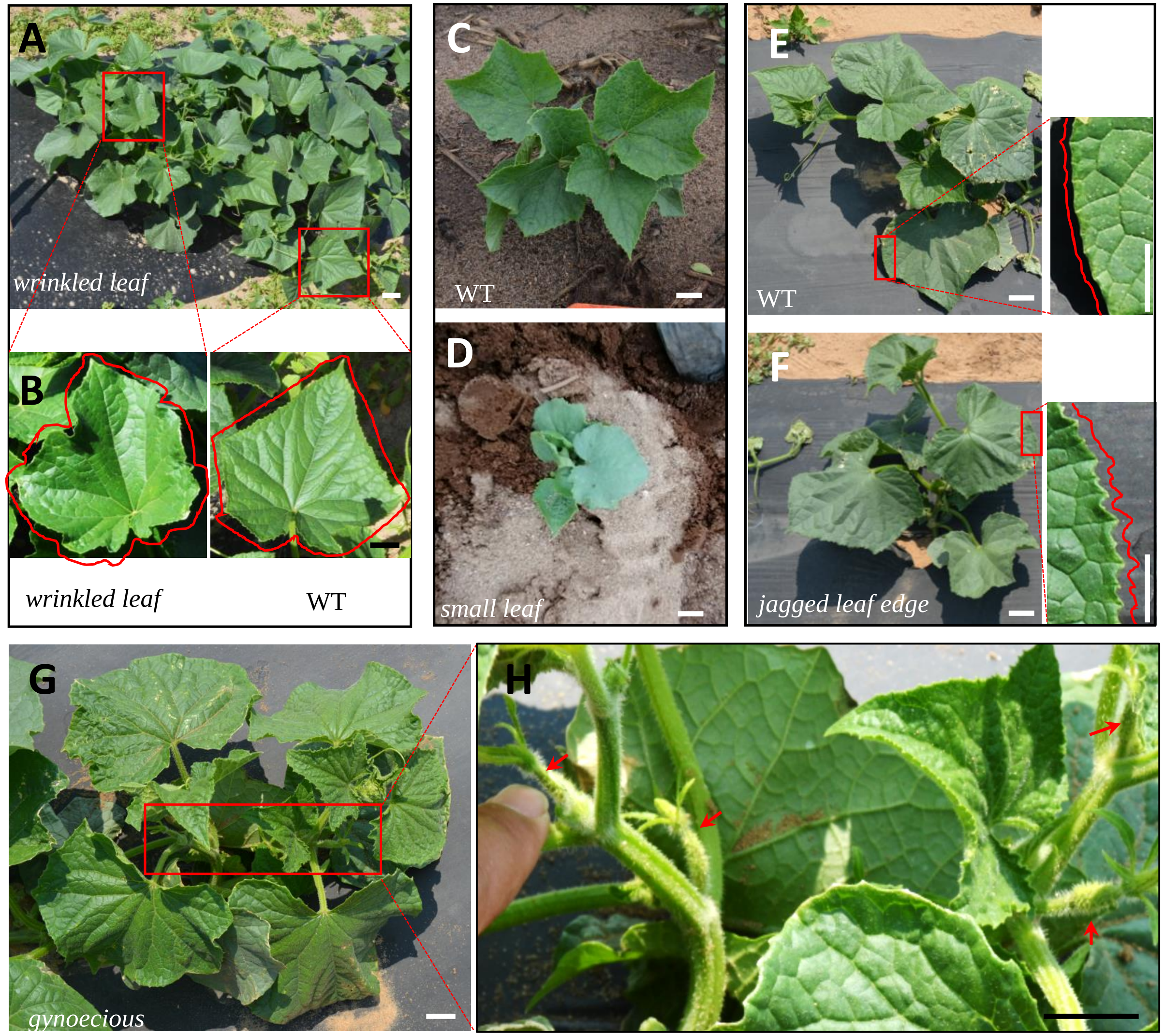

Figure S9. Examples of more putative T-DNA insertion mutants isolated from PS76-LL-OE T_1 transgenic plants. The putative mutants are *wrinkled leaf* (**A-B**), *small leaf* (**C-D**), *jagged leaf edge* (**E-F**) and *gynoecious* plants (**G-H**). WT = PS76. The red arrows indicate female flowers. Bar = 5.0 cm.
